# High-Temperature Reusability and In Situ Ceramization Mechanism of Alumina Fiber/Boron Phenolic Resin Composites Modified with ZrSi_2_ and TiB_2_

**DOI:** 10.3390/polym18101258

**Published:** 2026-05-21

**Authors:** Xiaobo Wan, Kaizhen Wan, Dongmei Zhao, Yiming Liu, Wenjing Cao, Zongyi Deng, Jian Li, Zhixiong Huang, Minxian Shi

**Affiliations:** 1 School of Materials Science and Engineering, Wuhan University of Technology, Wuhan 430070, China; 2 Hainan Institute, Wuhan University of Technology, Sanya 572000, China; 3 School of Automotive Materials, Hubei University of Automotive Technology, Shiyan 442002, China; 4 Hubei Provincial Research Institute of Advanced Aerospace Materials, Xiaogan 432100, China

**Keywords:** ceramizable composites, in situ ceramization, reusability, microstructural evolution

## Abstract

This research developed a ZrSi_2_-TiB_2_-modified alumina fiber/boron phenolic resin ceramizable composite intended to fulfill the criteria for high-temperature resistance, oxidation resistance, and structural load-bearing capacity in reusable thermal protection systems. The composite exhibits a low thermal conductivity of 0.405 W·m^−1^·K^−1^, a reduced density of 2.11 g·cm^−3^, and a high mass retention rate of 89.45% after heat treatment at 1200 °C in air. During thermal cycling at 1200 °C with a 30 min dwell time, it consistently demonstrates excellent stability, mass retention, and mechanical properties, indicating its potential for applications in reusable thermal protection systems. Following 20 cycles, the variation in length and width remains below 0.6%, the mass retention surpasses 80%, and the flexural strength remains above 20 MPa after 15 cycles. Microstructural evolution and thermodynamic analysis disclose that the in situ ceramization reaction of ZrSi_2_ and TiB_2_ consumes oxygen, inhibits oxygen diffusion, and fills pores and microcracks with oxidation products (SiO_2_ and B_2_O_3_), thereby forming self-healing and densifying phases. This synergistic mechanism of self-healing and densification ensures the reusability of the composite. The research illustrates the performance evolution patterns and strengthening mechanisms of the composite under extreme thermal conditions, confirming its outstanding performance in repeated usage evaluations.

## 1. Introduction

As reusable spacecraft are designed to operate at increasingly high flight speeds, they are exposed to progressively more severe aerothermal environments, which impose stricter requirements on thermal protection systems (TPS) [1,2,3,4]. Most existing thermal protection materials (TPMs) are primarily designed for single-use missions and therefore struggle to meet the urgent demands of next-generation spacecraft, particularly high-frequency reuse and low maintenance costs [5]. Consequently, the design of reusable thermal protection systems is imperative for the advancement of reusable spacecraft technology [6,7,8]. As key constituents of these systems, thermal protection materials require rapid improvement and innovation to meet reusable spacecraft requirements for lightweight design, high-temperature resistance, oxidation resistance, and repeated service [9,10].

Ablative thermal protection materials (ATPMs) are widely used in large-area TPS because of their low density, short fabrication cycles, and cost-effectiveness [6,11,12]. However, traditional composites such as high-silica–oxygen fiber/phenolic resin composites (HSF/Ph), quartz fiber/phenolic resin composites (QF/Ph), and carbon fiber/phenolic resin composites (CF/Ph) are generally limited in their reuse capability under high-temperature exposure. Although resin matrix modification can partially improve ablation resistance [13,14], Ding et al. [15] obtained an average flexural strength of 17.08 MPa for nano-ZrSi_2_-modified QF/Ph composites after 20 cycles of 10 min ablation at 1200 °C. Nonetheless, the primary silica constituent in high-silica fibers and quartz fibers undergoes significant crystalline precipitation at high temperatures [16,17], leading to structural defects and rapid deterioration of strength. Consequently, composites based on these fibers typically exhibit insufficient high-temperature mechanical properties, thus failing to meet the high-temperature load-bearing standards required for reusable spacecraft.

In contrast to high-silica fibers and quartz fibers, carbon fibers do not encounter high-temperature crystallization issues; however, they undergo degradation and oxidation above 450 °C. This oxidation results in catastrophic mechanical failure due to the proliferation of defects [18,19,20]. Oxidation of carbon fibers can be mitigated through matrix modification or fiber coating techniques, thereby enhancing the high-temperature load-bearing capacity of composite materials [21,22,23]. Zhang et al. [24] reported achieving a flexural strength of 66.91 MPa in MoSi_2_-modified carbon fiber/boron phenolic resin (CF/BPR) composites after 20 min heat treatment at 1500 °C. The application of matrix modification and fiber coating methods is frequently synergized to improve the high-temperature oxidation resistance of CF/BPR composites [25]. For example, Huang et al. [26] obtained a flexural strength of 41.8 MPa in Ti_3_SiC_2_ and CaB_6_ co-modified aluminum-coated carbon fiber/boron phenolic resin composites (ACF/BPR) after 20 min of heat treatment at 1400 °C. Although CF/BPR exhibits high flexural strength after a single high-temperature treatment, microstructural characterization indicates irreversible oxidative damage to both the fibers and the matrix consequent to thermal exposure [27,28]. This cumulative damage impedes the capability of carbon fiber phenolic resin composites to achieve genuine long-term, multi-cycle reuse [29].

In contrast, alumina fiber (Al_2_O_3_f)-reinforced composites have attracted considerable research attention due to their exceptional high-temperature performance [30]. Al_2_O_3_f not only exhibits low thermal conductivity and corrosion resistance [31], but also demonstrates exceptional chemical stability in high-temperature oxygen-rich environments [32,33], compensating for the propensity of CF to oxidize. Chang et al. [34] employed Al_2_O_3_f-hybridized CF to fabricate Al_2_O_3_f-CF/BPR composites, significantly enhancing oxidation resistance and high-temperature load-bearing capacity. However, CF reinforcements remain susceptible to progressive oxidation and failure during repeated usage cycles.

Overall, most existing studies on resin-based thermal protection composites have focused primarily on single-use applications, whereas their development for reusable service is still hindered by several critical issues, including the susceptibility of resin matrices to severe pyrolysis at elevated temperatures and the tendency of reinforcing fibers to undergo oxidation or crystallization during thermal exposure. To address these limitations and meet the requirements for reusable thermal protection materials, boron phenolic resin (BPR) was selected as the matrix for its superior high-temperature performance, while alumina fibers (Al_2_O_3_f) were used as the reinforcement owing to their excellent oxidation resistance and low tendency to crystallize. Based on this material design, Al_2_O_3_f-reinforced BPR composites (Al_2_O_3_f/BPR) were fabricated, and ZrSi_2_ and TiB_2_ were introduced as ceramic fillers to further enhance their ablation resistance [35,36]. The temperature of 1200 °C was selected as a representative high-temperature evaluation condition for large-area thermal protection structures in non-stagnation regions and lies within the engineering-relevant temperature range for polymer-based thermal protection materials [6]. In addition, a dwell time of 30 min was adopted to ensure that phenolic-based materials exposed to air could sufficiently undergo residual volatile release, char-layer oxidation, and pore/crack evolution, thereby yielding stable and comparable results in terms of mass loss and residual morphology, while also enabling an assessment of shape-retention capability under prolonged thermal exposure [6,37]. Accordingly, this work systematically investigates the reusability of Al_2_O_3_f/BPR composites under repeated heat-treatment cycles at 1200 °C for 30 min each, with particular emphasis on the influence of in situ ceramization during repeated thermal exposure, as well as the associated microstructural evolution and failure mechanisms.

## 2. Experiment

### 2.1. Raw Materials

Boron phenolic resin (THC-400, with boron content of 7 wt%) was utilized as the matrix material, supplied by Shaanxi Taihang Fire Retardant Polymer Co., Ltd. (Xi’an, China). Alumina fiber fabric (W-C150B1-370, with alumina content of 85 wt% and silica content of 15 wt%) served as the reinforcing material, supplied by Shanghai Rongrong New Material Technology Co., Ltd. (Shanghai, China). ZrSi_2_ particles (with a purity of 99.5% and an average particle size of 3 μm) were provided by Shanghai Chaowei Nano Technology Co., Ltd. (Shanghai, China). TiB_2_ particles (with a purity of 98% and particle size ranging from 4 μm to 8 μm) were supplied by Shanghai Aladdin Biochemical Technology Co., Ltd. (Shanghai, China). Both types of particles served as ceramic fillers. Anhydrous ethanol was obtained from Sinopharm Chemical Reagent Co., Ltd. (Shanghai, China).

### 2.2. Preparation of Ceramizable Composites

**Figure 1:**
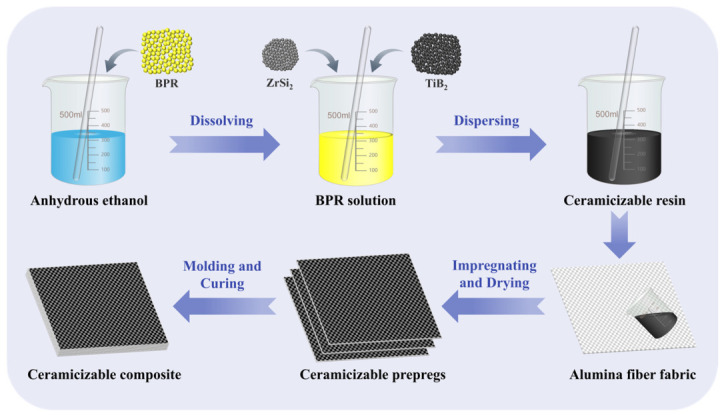
Schematic diagram of ceramizable composite preparation.

The preparation process for ceramizable composites is illustrated in Figure 1. Boron phenolic resin (BPR) is subjected to grinding followed by complete dissolution in an equal mass of anhydrous ethanol at 80 °C to produce a boron phenolic resin solution. According to the Z_20_T_25_ ratio specified in Table 1, ZrSi_2_ and TiB_2_ particles are dispersed into the resin solution. After 30 min of mechanical stirring to ensure uniform mixing, a ceramizable resin solution is obtained and evenly coated onto both surfaces of the alumina fiber fabric.

Following solvent evaporation at room temperature, the ceramizable prepreg is produced. The prepreg layers are then stacked within a metal mold and subjected to compression molding using a flat plate vulcanizer, following a predefined curing process to manufacture the ceramizable composite. The curing process involves preheating the metal mold with the impregnated fabric to 120 °C for 30 min to volatilize residual ethanol. Subsequently, the temperature is then raised to 180 °C and held for 25 min, after which a pressure of 10 MPa is applied. Temperature and pressure are sustained for 120 min. Ultimately, the temperature is increased to 200 °C and held for 60 min to complete the curing process. Furthermore, to examine the influence of incorporating ZrSi_2_ and TiB_2_ on the properties of the composite, a control sample without ceramic fillers is prepared according to the Z_0_T_0_ ratio specified in Table 1.

### 2.3. Oxyacetylene Ablation Test

In accordance with GJB 323A-96, the oxyacetylene ablation test was used to evaluate the ablation resistance of composites. Samples measuring 30 mm in diameter and (10 ± 0.1) mm thick were tested under the following two conditions: Condition 1: Heat flux density of 4186 kW·m^−2^, ablation duration of 30 s; Condition 2: Heat flux density of 1500 kW·m^−2^, ablation time of 60 s. During testing, the distance between the sample and the nozzle tip was fixed at (10 ± 0.2) mm, with an ablation angle of 90°. Linear ablation rate (*LAR*) and mass ablation rate (*MAR*) were used to evaluate ablation resistance of the composite quantitatively, calculated according to Equations (1) and (2), respectively:

(1) $$LAR=\frac{∆d}{t}=\frac{d_{1}-d_{2}}{t}$$

(2) $$MAR=\frac{∆m}{t}=\frac{m_{1}-m_{2}}{t}$$

The variable *t* indicates the ablation duration. The parameters *m*_1_ and *m*_2_ correspond to the mass of the composite prior to and following ablation, respectively. The parameters *d*_1_ and *d*_2_ denote the thickness of the ablation zone within the composite before and after the process, respectively.

### 2.4. High-Temperature Heat Treatment in Muffle Furnaces

To evaluate the oxidation resistance, high-temperature load-bearing capacity, and reusability of the ceramizable composites, they were subjected to high-temperature heat treatment in a muffle furnace. The composites were machined into 60 × 15 × 3 mm^3^ flexural samples and placed in an alumina crucible. After the muffle furnace reached 1200 °C, the alumina crucible was then transferred to the furnace. Once the temperature reached 1200 °C, it was dwelled for 30 min. Following the holding period, the crucible was removed, and the sample was allowed to cool naturally to room temperature in air. Subsequently, the cooled sample was returned to the muffle furnace at 1200 °C, and the aforementioned heat treatment process was repeated to produce flexural samples subjected to 1, 5, 10, 15, and 20 heat treatment cycles, respectively.

### 2.5. Characterization

The thermal stability of the prepared composite in an air atmosphere was assessed using a simultaneous thermal analyzer (NETZSCH STA 2500, NETZSCH Group, Selb, Germany). The test conditions involved heating from room temperature to 1500 °C at a rate of 10·°C min^−1^. The thermal conductivity of the composite was measured employing a thermal conductivity tester (QTM-500, KYOTO ELECTRONICS MANUFACTURING Co., Ltd., Kyoto, Japan) in accordance with GB/T 10297-2015, with the heating current set between I^2^ = 1.0–2.0 A^2^. The flexural strength of samples, both before and after high-temperature heat treatment, was evaluated using an electronic universal testing machine (RGM-2100, Shenzhen Rigel Company, Shenzhen, China) in a three-point flexural mode, in accordance with GB/T 1449-2005. Microstructural analyses of sample surfaces and fracture surfaces, both before and after high-temperature heat treatment, were conducted using scanning electron microscopy (SEM, TESCAN MIRA LMS, TESCAN, Brno, Czech Republic) and X-ray energy-dispersive spectrometry (EDS, Oxford Smartedx, Oxford Intelligent (Hangzhou) Technology Co., Ltd., Hangzhou, China), which enabled the analysis of element distribution and content within the samples. The phase composition of the composite was characterized using an X-ray diffractometer (XRD, Bruker D8 Advance, Bruker AXS, Karlsruhe, Germany), with scanning parameters set at a rate of 5°·min^−1^ over a scanning range of 10° to 80° (2θ).

## 3. Results and Discussion

### 3.1. Thermal Performance and Ablation Resistance

To assess the thermal insulation performance of the composite, its thermal conductivity was measured (as detailed in Table 2). The thermal conductivity of Z_0_T_0_ devoid of ceramic fillers was observed to be 0.268 W·m^−1^·K^−1^. Owing to the filling effect of ZrSi_2_ and TiB_2_, defects such as micro-pores formed during the curing process were mitigated, thereby decreasing the interfacial thermal resistance. As a result, the thermal conductivity of Z_20_T_25_ increased to 0.405 W·m^−1^·K^−1^. While the incorporation of ceramic fillers enhanced the thermal conductivity of the composite, it nonetheless remained considerably lower than that of carbon fiber-reinforced carbon–phenolic resin composite (CF/CPR), carbon/carbon composites (C/C), and carbon fiber-reinforced ultra-high-temperature ceramic matrix composites (CF/UHTCM) [38,39,40]. This phenomenon is primarily attributed to the low thermal conductivity of Al_2_O_3_f [31], which confers excellent thermal insulation performance to the composite.

**Figure 2:**
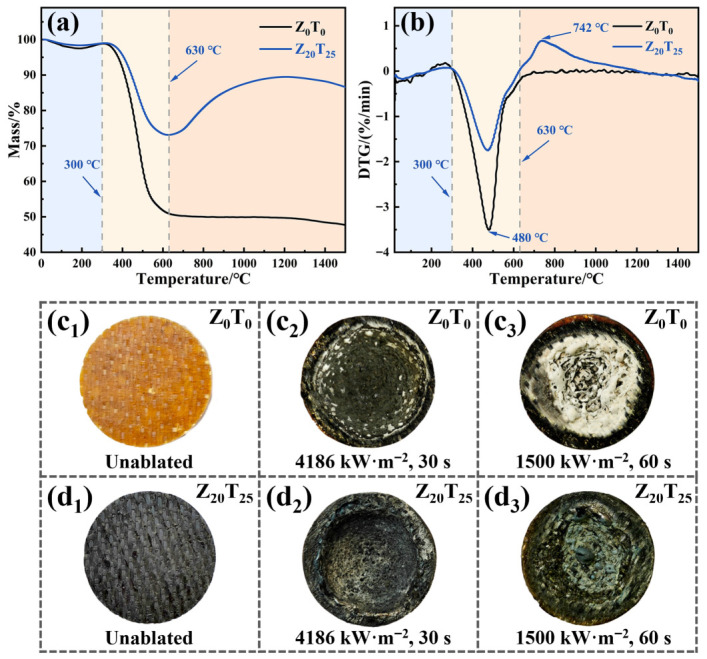
(**a**) TG curve, (**b**) DTG curve, macrographs of samples: (**c**) Z_0_T_0_ and (**d**) Z_20_T_25_ before and after oxyacetylene ablation ((**c_1_**) is Z_0_T_0_ before ablation, (**c_2_**) is Z_0_T_0_ after 30 s of ablation at 4186 kW·m^−2^, (**c_3_**) is Z_0_T_0_ after 60 s of ablation at 1500 kW·m^−2^, (**d_1_**) is Z_20_T_25_ before ablation, (**d_2_**) is Z_20_T_25_ after 30 s of ablation at 4186 kW·m^−2^, and (**d_3_**) is Z_20_T_25_ after 60 s of ablation at 1500 kW·m^−2^).

To further investigate the effects of ZrSi_2_ and TiB_2_ on the thermal stability of Al_2_O_3_f/BPR, thermogravimetric analysis (TG) and differential thermal gravimetric analysis (DTG) were conducted, and the results are presented in Figure 2a,b.

The thermal decomposition and oxidation process of Z_0_T_0_ can be categorized into three distinct stages. The initial stage (<300 °C) exhibits a minor mass reduction of 1.1%, attributed to the volatilization of adsorbed water and the release of free phenols from the sample. The subsequent stage (300–630 °C) is characterized by intense thermal decomposition and oxidation, with a mass loss of 47.9% and a peak mass loss rate observed at 480 °C (Figure 2b). The mass loss during this phase mainly results from the thermal decomposition of BPR, which releases small molecules such as CH_4_, CO, H_2_O, and phenol, in addition to the oxidation of the resultant pyrolytic carbon (PyC). During the final stage (630–1500 °C), the TG curve displays a gradual decline, with a total mass loss of only 3.3%. The mass loss in this stage is primarily due to the volatilization of B_2_O_3,_ the oxidation product.

The pyrolysis process of Z_20_T_25_ containing ceramic fillers closely resembles that of Z_0_T_0_, also comprising three stages. Below 300 °C, the TG curves of both samples are essentially indistinguishable, each exhibiting a mass loss of 1.1%. This reduction in mass at this stage is ascribed to the volatilization of adsorbed water. Within the range of 300 °C to 630 °C, Z_20_T_25_ exhibits significant mass loss, following a trend consistent with Z_0_T_0_. However, its mass loss is only 25.8%, representing a 22.1% reduction compared to Z_0_T_0_. This decreased rate of mass loss is primarily due to the oxidation of the ceramic fillers in Z_20_T_25_, which consumes oxygen and thereby reduces the partial pressure of oxygen. Simultaneously, the produced oxides deposit onto the resin matrix and fiber surfaces, shielding contact between BPR and oxygen. This synergistic mechanism of oxygen depletion and the formation of an oxygen barrier effectively inhibits BPR pyrolysis and PyC oxidation, resulting in a notable decrease in mass loss. As illustrated in Figure 2b, although both samples reached their maximum mass loss rates at 480 °C, the rate for Z_20_T_25_ remained consistently lower than that of Z_0_T_0_, supporting the aforementioned mechanism. During the high-temperature stage from 630 °C to 1500 °C, their thermal behaviors demonstrate significant divergence. In contrast to the gradual decrease observed in the TG curve of Z_0_T_0_, the TG curve of Z_20_T_25_ initially exhibits a rapid increase before gradually declining. This behavior is attributed to the ceramic fillers incorporated into Z_20_T_25_, which undergo ongoing oxidation, resulting in mass gain at high temperature. Its rate of weight gain surpasses the mass loss caused by B_2_O_3_ volatilization, resulting in an increase, rather than a decrease, in the residual mass of the sample. The DTG curve indicates that the oxidation mass gain rate of the ceramic fillers peaks at 742 °C. As most of the ceramic fillers are oxidized, the oxidation rate of the remaining fillers gradually diminishes, attenuating the mass gain trend of the sample. Consequently, the residual mass begins to decrease gradually.

The ablation resistance of the composite was assessed through oxygen-acetylene ablation tests. Figure 2c,d illustrate the macroscopic morphology of the samples prior to and following ablation. Three samples were tested for each group, and the mean values and standard deviations of the detailed ablation data are provided in Table 3. As depicted in Figure 2(c_1_,d_1_), the unablated Z_0_T_0_ sample presents an orange-yellow appearance, whereas the Z_20_T_25_ sample appears grayish-black. Both samples display smooth surfaces with identifiable Al_2_O_3_f patterns.

Following an ablation duration of thirty seconds at a heat flux density of 4186 kW·m^−2^, the surface of Z_0_T_0_ devoid of ceramic fillers (Figure 2(c_2_)) turned black due to oxidation, characterized by the formation of a prominent crater at the center of ablation surrounded by a region of white reaction products. Under these extreme conditions, the flame temperature at the ablation center attained 3000 °C [41,42], significantly surpassing the melting point of Al_2_O_3_ (2072 °C). This resulted in rapid oxidation pyrolysis of the BPR, with the melted Al_2_O_3_f being entirely eroded by the flame gases, leaving behind small quantities of partially melted white Al_2_O_3_f at the crater periphery. Owing to substantial loss of resin and fibers, Z_0_T_0_ demonstrated a high *LAR* and *MAR* of 0.1350 mm·s^−1^ and 0.0652 g·s^−1^, respectively. Under identical ablation conditions, the Z_20_T_25_ sample (Figure 2(d_2_)), which incorporates ceramic fillers, also exhibited pitted surface morphology post-ablation. However, the pits were marginally smaller, featuring pale white centers with no significant exposure of Al_2_O_3_f. This phenomenon is attributable to the oxidation of ceramic fillers during ablation, with oxidation products adhering to the sample surface, thereby offering partial protection to the resin matrix and fibers, and delaying their pyrolysis and melting processes. Nonetheless, the data presented in Table 3 indicate that at this heat flux density, the *LAR* of Z_20_T_25_ (0.1363 mm·s^−1^) is nearly identical to that of Z_0_T_0_. Experimental results suggest that at exceedingly high temperatures, the *LAR* is predominantly governed by the melting of Al_2_O_3_, with negligible influence exerted by the ceramic fillers. Conversely, the *MAR* of Z_20_T_25_ decreases to 0.0570 g·s^−1^, which is attributed to the oxidative protection conferred by the ceramic fillers and their associated oxidative mass gain.

Considering that the differences between Z_0_T_0_ and Z_20_T_25_ at a heat flux density of 4186 kW·m^−2^ were not statistically significant, the heat flux density was modified to 1500 kW·m^−2^, and the ablation duration was extended to 60 s. The post-ablation morphologies are presented in Figure 2(c_3_,d_3_). Under these conditions, a pit approximately one-third the size of that in Figure 2(c_2_) manifests at the center of Z_0_T_0_ (Figure 2(c_3_)), with the sample surface remaining comparatively flat. The extensive white fibrous network surrounding the pit corresponds to exposed Al_2_O_3_f. This phenomenon occurs because the reduced heat flux density diminishes the sample temperature; however, the ablation center remains heated sufficiently to reach the melting point of Al_2_O_3_. Consequently, fibers within the ablation center melt and are eroded, forming the pit. While temperatures in the surrounding areas are insufficient to melt the fibers, they still induce rapid oxidation and pyrolysis of the resin, leaving the exposed fiber network intact. Compared to the high *LAR* (0.1350 mm·s^−1^) at 4186 kW·m^−2^, the *LAR* of Z_0_T_0_ at 1500 kW·m^−2^ significantly decreased to 0.0343 mm·s^−1^. This reduction is primarily attributed to a substantial decrease in molten Al_2_O_3_f, while the sample thickness remained relatively stable. Despite the reduction in fiber melting, the resin still undergoes rapid pyrolysis at high temperature. The extended ablation time of 60 s increases the pyrolysis mass of the resin, resulting in only a slight decrease in the *MAR* of Z_0_T_0_ from 0.0652 g·s^−1^ to 0.0502 g·s^−1^.

At an identical heat flux density of 1500 kW·m^−2^, the Z_20_T_25_ sample (Figure 2(d_3_)) also demonstrated pitting at the center following ablation. Nevertheless, its surface was covered with a grayish-green layer, and no extensive fiber exposure was observed. Z_20_T_25_ exhibited a *LAR* of 0.0317 mm·s^−1^, marginally lower than that of Z_0_T_0_ under comparable test conditions. However, its *MAR* experienced a significant reduction to 0.0198 g·s^−1^, accounting for only 62.5% of the *MAR* value of Z_0_T_0_. This notable enhancement can be primarily attributed to the synergistic effect of ceramic fillers, which undergo oxidation at high temperature, consuming oxygen in the process. The resulting dense oxide layer effectively prevents oxygen contact with the resin and fibers. This mechanism substantially suppresses the oxidative pyrolysis of BPR. Simultaneously, the fillers gain additional mass through oxidation, collectively contributing to the marked decrease in *MAR*.

In the context of thermal protection materials, the significance of exceptional ablation resistance is equally complemented by robust thermal insulation performance. During the oxyacetylene ablation test, the average backface temperature of the composite subjected to ablation was measured utilizing thermocouples, with the results detailed in Table 3. After 30 s of ablation at a heat flux density of 4186 kW·m^−2^, the backface temperature of Z_0_T_0_ was recorded at 40.1 °C, whereas that of Z_20_T_25_ was 52.9 °C. Following 60 s of ablation at a heat flux density of 1500 kW·m^−2^, the backface temperature of Z_0_T_0_ reached 65.8 °C, in comparison to 71.0 °C for Z_20_T_25_. These findings suggest that incorporating ceramic fillers into the composite enhances its thermal conductivity, which subsequently reduces its thermal insulation performance. This observation aligns with the results obtained from previous thermal conductivity tests. Although Z_0_T_0_ exhibits superior thermal insulation performance, its ablation rate remains prohibitively high. Overall, the inclusion of ceramic fillers markedly improves the ablation resistance of the composite, with Z_20_T_25_ demonstrating superior overall performance.

### 3.2. High-Temperature Heat Treatment in Muffle Furnaces and the Evolution of Dimensions and Mechanical Strength

**Figure 3:**
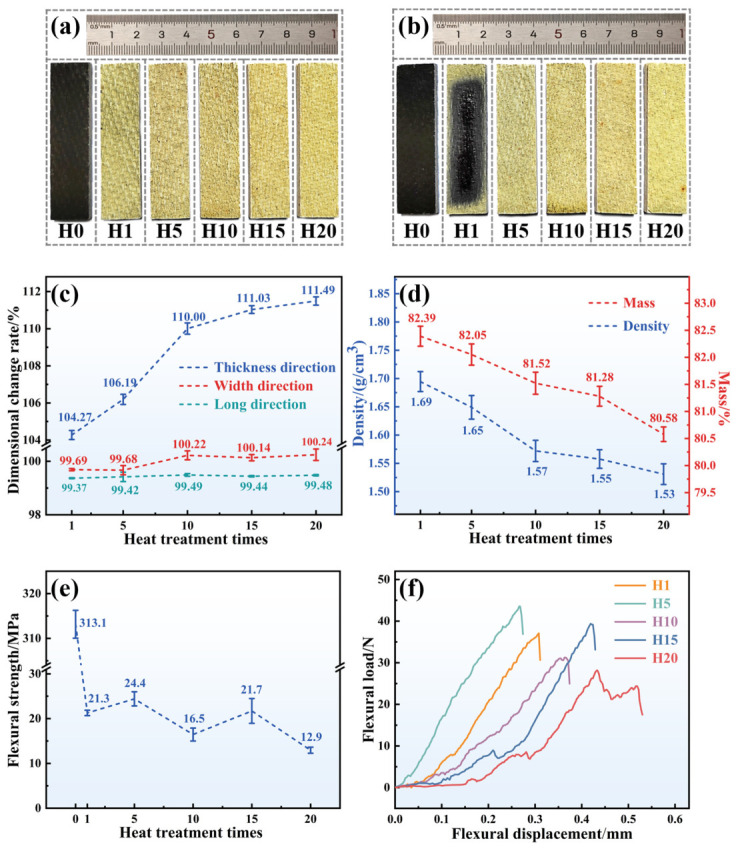
Z_20_T_25_ flexural sample muffle furnace after repeated thermal cycling: (**a**) front surface macrostructure, (**b**) backface macrostructure, (**c**) dimensional change rate, (**d**) mass retention rate and density, (**e**) flexural strength, and (**f**) load–displacement curve.

To evaluate the reusability characteristics of composites, flexural samples were subjected to thermal treatment in a muffle furnace maintained at 1200 °C for 30 min. Following cooling to room temperature after removal, the procedure was repeated, involving multiple successive cyclic heat treatments. Due to the limited thermal stability and ablation resistance of Z_0_T_0_, only Z_20_T_25_, which exhibits superior overall performance, was selected for repeated thermal treatments. Samples subjected to 1, 5, 10, 15, and 20 heat treatments were designated as H1, H5, H10, H15, and H20, respectively (the untreated sample was designated as H0; macroscopic morphology is illustrated in Figure 3a,b).

As illustrated in Figure 3a, the initial sample H0 (depicted in black) rendered almost entirely yellow following the first heat treatment (H1). This transformation resulted from the pyrolysis of BPR and the oxidation of ceramic fillers. Fine-scale Al_2_O_3_f patterns were observable on the surface of H1; however, they were not exposed. As the number of thermal cycles increased, the residual black regions progressively diminished. By H15 and H20, the surface had completely adopted a yellow coloration, signifying complete oxidation of the ceramic fillers. Simultaneously, microcracks began to appear on the sample surface. Notably, slight delamination was observed on the side of the H20 sample, potentially leading to its diminished flexural strength. The pattern of color change on the reverse side of the sample was consistent with that of the front (Figure 3b). Consistent color changes were shown on both the front and reverse sides of the sample (Figure 3b). Nevertheless, owing to contact with the crucible, oxygen supply to the backside of the sample was relatively inadequate. The central region of H1 retained a grayish-black hue due to incomplete oxidation. As the number of thermal cycles increased, the back side of H5 turned entirely yellow, and the morphologies of the backsides of H10, H15, and H20 became essentially analogous to their front sides. A comparison of macroscopic morphology revealed that, after multiple high-temperature heat treatments, the samples exhibited no significant changes in shape or surface spalling beyond color alterations. This observation indicates that Z_20_T_25_ possesses excellent dimensional stability.

Regarding reusable thermal protection materials, it is crucial to verify their dimensional stability and mass consistency across successive applications. Therefore, the dimensions along the three orthogonal axes (length, width, and thickness) along with their corresponding masses, were recorded throughout heat treatment in a muffle furnace. The rates of dimensional variation and mass retention are depicted in Figure 3c,d, respectively.

As demonstrated in Figure 3c, during the initial 20 heat treatment cycles, the length and width dimensions of the samples showed remarkable stability. The rate of dimensional change in the longitudinal direction consistently remained below 0.2%, whereas in the transverse direction, it persisted below 0.6%. These measurements correspond to the axial orientation of the Al_2_O_3_f. The intrinsic dimensional stability of the fibers guarantees the overall dimensional stability of the composite. Conversely, the rate of change in thickness was markedly higher, increasing from 104.27% at H1 to 111.49% at H20, representing a 7.22% increase. Significantly, between H5 and H10, the dimensional change rate in thickness increased by 3.81%, despite only five additional heat treatment cycles. The composite is produced through stacking and pressing multiple layers of prepreg, which is the primary cause of the significant dimensional change in the thickness direction. After repeated long-term high-temperature treatments, the BPR undergoes oxidative pyrolysis, while the ZrSi_2_ and TiB_2_ ceramic fillers expand volumetrically upon oxidation. Notably, the B_2_O_3_ produced from TiB_2_ oxidation demonstrates high volatility above 1000 °C [43], leading to the interlaminar bond degradation and slight delamination, which subsequently results in a significant increase in thickness.

Calculations indicate that the initial density of Z_20_T_25_ is 2.11 g·cm^−3^. As shown in Figure 3d, both the mass retention rate and density of the sample gradually decrease with increasing heat treatments, with the mass retention rate consistently remaining above 80%. From H1 to H20, the mass retention rate declined by only 1.81%, and the density decreased by merely 0.16 g·cm^−3^, further demonstrating the mass stability of Z_20_T_25_ during repeated heat treatments. Notably, although the mass retention rate exhibits a uniform decline with increasing heat treatments, the density change occurs in two distinct phases: Phase I (H1 to H10) shows a significant decrease in density, particularly a 3.79% drop between H5 and H10; Phase II (H10 to H20) experiences only a 1.90% reduction in density over 10 additional heat treatments. When examined in conjunction with the dimensional change data illustrated in Figure 3c, it becomes apparent that the decrease in density, which occurs concurrently with minimal variations in mass, is predominantly attributable to the expansion of the sample dimensions—particularly its thickness. Excellent dimensional stability and consistent mass retention are vital for the high reliability of reusable thermal protection materials, while the load-bearing capacity during repeated testing cycles holds equal significance. Accordingly, flexural tests were performed on samples subjected to different heat treatment cycles to assess their long-term oxidation resistance and high-temperature load-bearing capacity.

As illustrated in Figure 3e, the unheated Z_20_T_25_ (H0) exhibits a flexural strength of 313.1 MPa, signifying an outstanding load-bearing capacity at high temperature. After one heat treatment cycle (H1), the flexural strength substantially diminishes to 21.3 MPa. Subsequent heat treatments, conducted four additional times on H1 to yield H5, result in a slight rebound in flexural strength to 24.4 MPa. Further increments in the number of heat treatment cycles result in a decline in flexural strength to 16.5 MPa for H10, followed by a recovery to 21.7 MPa for H15. In contrast, H20, after twenty heat treatments, underwent a significant decrease in strength to 12.9 MPa. This trend indicates that flexural strength does not decrease linearly with increasing heat treatment cycles, but instead exhibits notable fluctuations. This phenomenon suggests that the thermal evolution is dominated by complex interactions among constituents, rather than being limited to individual processes like resin pyrolysis or filler oxidation. These transformations and their resulting products have a significant impact on the mechanical properties of the composites.

Figure 3f illustrates the load–displacement curves of samples following repeated heat treatment. The load–displacement curves for samples H1 and H5 are nearly linear, reaching a peak before experiencing a precipitous decline in load, thereby exemplifying typical brittle fracture behavior. Subsequent samples H10, H15, and H20 also demonstrated brittle fracture; however, their load–displacement curves before failure did not conform to a single straight line. Instead, they displayed a sawtooth pattern, a phenomenon that was markedly evident in H15 and H20. This suggests that, after multiple heat treatments, the interlaminar bonding within sample composites fabricated from multilayer prepregs diminished in cohesion. Under applied flexural loads, delamination occurred, manifesting as the sawtooth pattern observed in the load–displacement curve. The considerable delamination within the composite, coupled with dimensional expansion in the thickness direction, ultimately caused the flexural strength of H20 to decline sharply to 12.9 MPa.

### 3.3. Microstructural Evolution of Composite Surfaces

**Figure 4:**
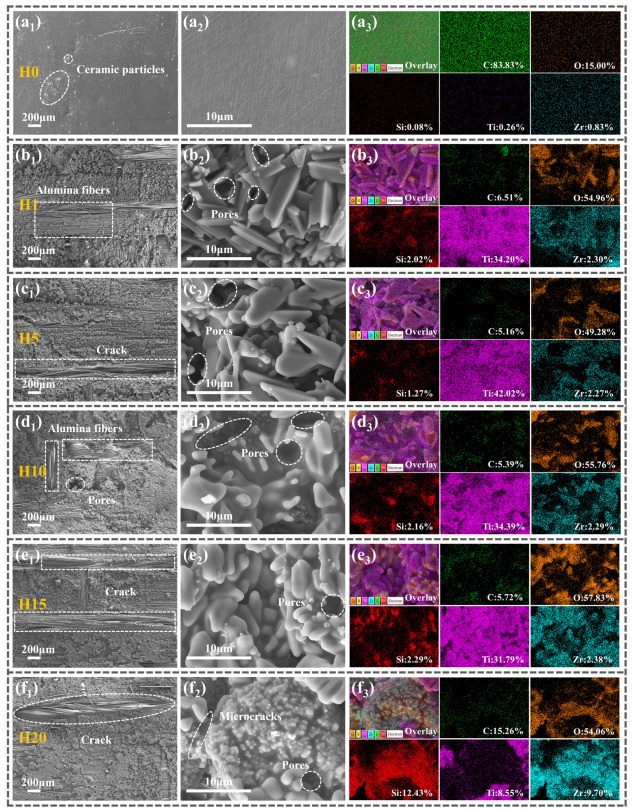
(**a_1_**–**a_3_**) H0, (**b_1_**–**b_3_**) H1, (**c_1_**–**c_3_**) H5, (**d_1_**–**d_3_**) H10, (**e_1_**–**e_3_**) H15, and (**f_1_**–**f_3_**) H20 surface microstructures and EDS spectra (*x*_1_ shows a low-magnification image, *x*_2_ shows a high-magnification image, and *x*_3_ shows the EDS spectrum).

To investigate the causes of fluctuations in flexural strength in Z_20_T_25_ during repeated high-temperature heat treatment, the surface microstructure of samples H0–H20 was examined, and the elemental distribution and content in corresponding regions were analyzed, as illustrated in Figure 4.

Figure 4(a_1_–a_3_) illustrates the microstructure of the untreated Z_20_T_25_ (H0) sample. The low-magnification image (Figure 4(a_1_)) shows a smooth, flat surface with only minor protrusions of ceramic fillers, free from voids or other defects. The high-magnification image (Figure 4(a_2_)) further confirms its dense microstructure. This indicates that, under the curing process utilized in this research, Al_2_O_3_f, BPR, ZrSi_2,_ and TiB_2_ exhibit good compatibility, resulting in superior composite performance. Figure 4(a_3_) demonstrates a uniform distribution of elements without significant agglomeration, suggesting that the ceramic fillers are evenly dispersed within the BPR matrix. Energy dispersive X-ray spectroscopy analysis reveals that the surface of the sample is predominantly composed of BPR. The elements carbon (83.83%) and oxygen (15.00%), which constitute the highest proportions, both originate from BPR. Additionally, trace amounts of zirconium, silicon, and titanium from the ceramic fillers are uniformly distributed across the surface. Due to its low boron content and the difficulty in detection, its signal is not visible in the energy spectrum and is consequently not depicted in Figure 4.

Following a single heat treatment (H1), significant modifications in surface morphology and elemental composition were observed (Figure 4(b_1_–b_3_)). The surface became loose and porous (Figure 4(b_1_)), with extensive exposure of Al_2_O_3_f, attributable to high-temperature pyrolysis of BPR and oxidation of PyC. As illustrated in Figure 4(b_3_), the carbon content experienced an abrupt decline to 6.51%, while the oxygen content markedly increased to 54.96%. This reduction in carbon results from the volatilization of small molecules such as CH_4_ and CO during BPR pyrolysis, as well as the oxidation of PyC. The substantial consumption of BPR exposed and oxidized additional ceramic fillers, resulting in a relative increase in the contents of zirconium, silicon, and titanium. The oxidation process consumed oxygen and produced oxides, thereby increasing the elemental oxygen content within the material. Furthermore, Figure 4(b_3_) shows that the prismatic material predominantly consists of TiO_2_, an oxidation product of TiB_2_. These oxides form a coating on the substrate surface intended to inhibit oxygen diffusion; however, their large particle size and sharp prismatic morphology (Figure 4(b_2_)), coupled with the abundance of voids between particles, impede the formation of a dense protective layer. Consequently, their protective efficacy on BPR and PyC remains limited.

Four additional heat treatments of H1 produced H5, with microstructure and elemental composition illustrated in Figure 4(c_1_–c_3_). The low-magnification image (Figure 4(c_1_)) resembles H1, displaying a loose, porous structure with exposed fibers and cracks between fiber bundles. Energy dispersive spectroscopy (EDS) results are consistent with H1, indicating the chemical stability of the material during heat treatment. Nevertheless, the high-magnification image (Figure 4(c_2_)) demonstrates that the ceramic oxides here transition into smaller, disc-shaped structures. These conform more effectively to the surfaces of BPR and PyC, thereby offering enhanced protection. This morphological change in the ceramic oxide results from the high-temperature environment, causing high-surface-energy prismatic oxides to gradually convert into low-surface-energy lamellar oxides, while some larger particles decompose or break down into smaller particles. The refinement of the ceramic oxide serves to optimize stress distribution within the composite, reduce stress concentration, and improve interfacial bonding via crack deflection mechanisms. As a result, the flexural strength of H5 exhibits a slight increase compared to H1 (Figure 3e).

The microstructure and elemental distribution of the H10 surface showed no significant alterations; however, the exposed fiber area increased with progressive thermal cycles (Figure 4(d_1_)). Figure 4(d_2_) demonstrates that the ceramic oxide particles became more refined and were enveloped by a film-like substance, which was identified as amorphous SiO_2_ through elemental analysis. Notably, numerous interconnected pores emerged around the oxides. These pores predominantly resulted from the formation and volatilization of gas molecules such as B_2_O_3_, CH_4_, and CO at high temperatures. The increased porosity contributed to an increase in the number of defects within the material. As flexural strength is susceptible to defects, the strength of H10 experienced a significant reduction (Figure 3e).

The surface morphology of H15 (Figure 4(e_1_)) generally corresponds with that of H10, although the inter-fiber cracks are more conspicuous. Its high-magnification image (Figure 4(e_2_)) exhibits ceramic oxides as ellipsoidal particles, similarly coated with a glassy phase film. The principal distinction from H10 is the lack of large interconnected voids in the H15 sample. During subsequent heat treatment, the glass phase undergoes secondary viscous flow at high temperatures, refilling pores and microcracks. This process reduces surface energy and diminishes stress concentration. Furthermore, the refined oxide particles in H10 co-sinter with the glass phase, forming the more stable ellipsoidal structure observed in H15. This contributes to enhanced geometric continuity of the composite, resulting in a subsequent increase in the flexural strength of H15 (Figure 3e).

The low-magnification image of the H20 sample (Figure 4(f_1_)) exhibits minimal alterations; however, the cracks between fiber bundles have further expanded. Its high-magnification image (Figure 4(f_2_)) exposes an irregular, coral-like morphology composed of numerous fine spherical protrusions clustered together, with lamellar oxides visible in the surrounding regions. Figure 4(f_3_) indicates a significant reduction in titanium content within this region, accompanied by a substantial increase in silicon and zirconium content. Integrating morphological and elemental data, it is inferred that the coral-like material predominantly consists of ZrSiO_4_. The combined microscopic morphologies of H1–H20 demonstrate that two varieties of ceramic oxides coexist on the surface of the heat-treated sample: one being a plate-like structure primarily consisting of TiO_2_ (observed in the H1–H15 region), and the other being a coral-like structure mainly composed of ZrSiO_4_ (observed in the H20 region).

### 3.4. Microstructural Evolution of the Fracture Surface Under Flexural Loading

**Figure 5:**
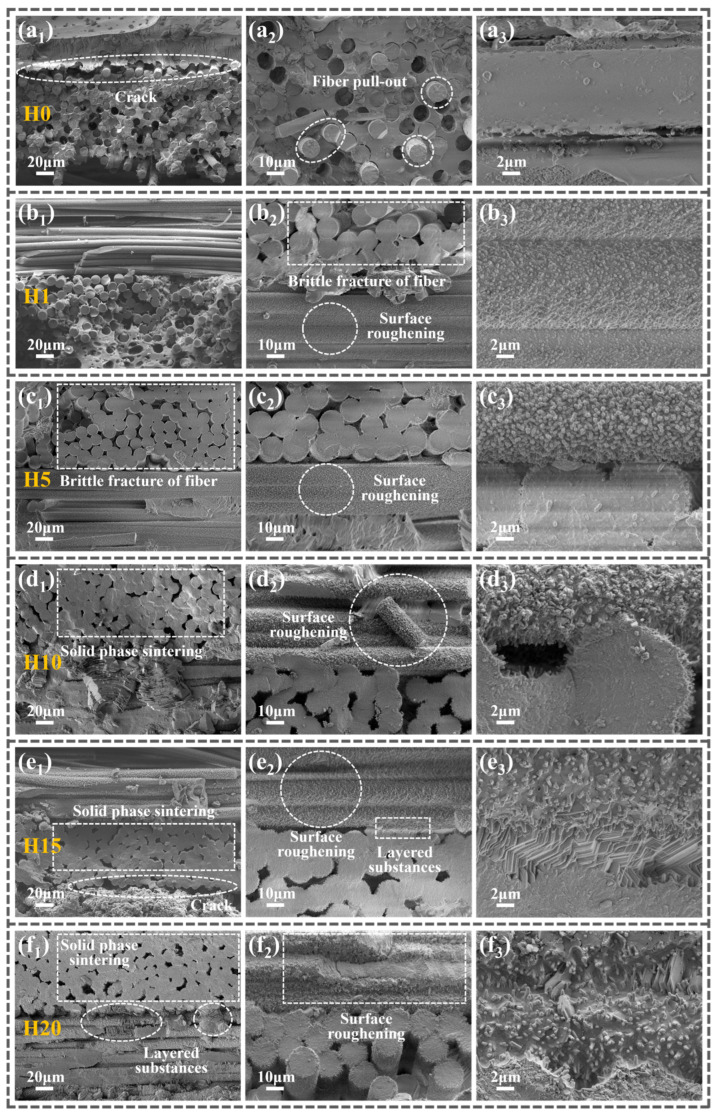
(**a_1_**–**a_3_**) H0, (**b_1_**–**b_3_**) H1, (**c_1_**–**c_3_**) H5, (**d_1_**–**d_3_**) H10, (**e_1_**–**e_3_**) H15, and (**f_1_**–**f_3_**) H20 microstructures of the flexural fracture surface (*x*_1_ shows a low-magnification image, *x*_2_ shows a high-magnification image, and *x*_2_ shows the surface morphology of the fiber).

**Figure 6:**
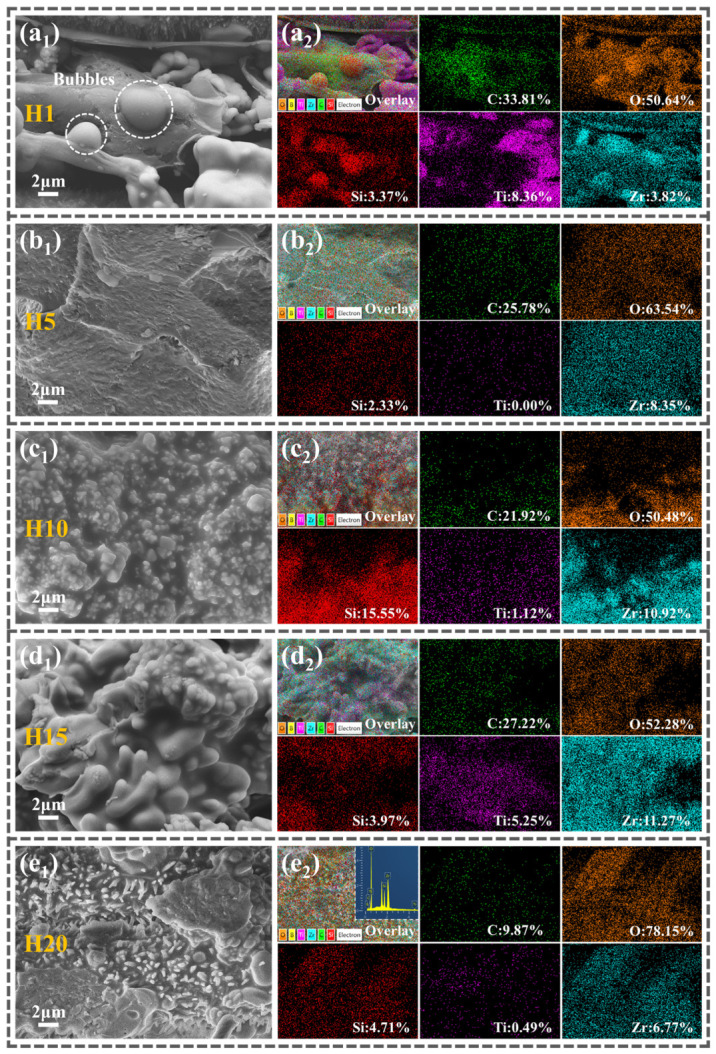
(**a_1_**,**a_2_**) H1, (**b_1_**,**b_2_**) H5, (**c_1_**,**c_2_**) H10, (**d_1_**,**d_2_**) H15, and (**e_1_**,**e_2_**) H20 flexural fracture surface EDS spectra (*x*_1_ represents the microstructure, and *x*_2_ represents the EDS spectrum).

Through the characterization and analysis of the microstructure of the H0–H20 surface, we have initially elucidated some potential reasons for the fluctuations in the flexural strength of Z_20_T_25_ during repeated heat treatment. To conduct a comprehensive analysis of the evolution mechanism of flexural strength in Z_20_T_25_ during repeated high-temperature heat treatment, additional examinations were performed on the fracture surface morphology (Figure 5) and element distribution (Figure 6) of the H0–H20 samples.

The microstructure of the H0 sample flexural fracture surface is illustrated in Figure 5(a_1_–a_3_). Continuously aligned Al_2_O_3_f forms an effective load-bearing network within the composite (Figure 5(a_1_)), supporting primary stresses under flexural loads. Furthermore, Figure 5(a_1_) reveals resin fragments adhering to the pulled-out fibers, indicating robust interfacial bonding between the fibers and matrix. This strong interfacial adhesion facilitates efficient stress transfer and additional energy dissipation through fiber pull-out behavior during ultimate fracture. Figure 5(a_2_) illustrates distinct fiber pull-out phenomena, which substantially enhance the flexural strength of the sample. The combination of continuous fiber orientation and excellent interfacial adhesion collectively ensures the superior flexural performance of the material (313.1 MPa).

The fracture surface (Figure 5(b_1_)) revealed that the interlaminar bonding retained its integrity, characterized by a smooth fracture plane and mirror-like fiber fracture edges, with no evidence of fiber pull-out. Exposure to elevated temperatures led to increased crystallinity of Al_2_O_3_f and caused surface erosion (Figure 5(b_2_,b_3_)) [44], which can readily induce stress concentration. This combination of factors resulted in the observed brittle fracture behavior. High-magnification images (Figure 6(a_1_)) revealed large voids within H1, where gases such as B_2_O_3_, CH_4_, and CO were trapped by the surface glass phase, resulting in numerous bubbles. The synergistic effects of Al_2_O_3_f hardening and embrittlement, surface erosion of the fibers, and stress concentration at defect sites resulted in a significant decrease in the strength of H1. Figure 6(a_2_) indicates that the carbon content on the fracture surface of H1 was 33.81%, markedly higher than the 6.51% observed on its surface (Figure 4(b_3_)), signifying that the BPR and PyC within H1 were effectively better protected.

The fracture morphology of H5 (Figure 5(c_1_,c_2_)) resembles that of H1, exhibiting tight interlamellar bonding, a smooth fracture surface, and brittle fracture characteristics. After four additional heat treatments, sintered necks formed at the contact points between adjacent fibers in H5 due to the flow of the glass phase (Figure 5(c_2_)) [44]. Concurrently, Al_2_O_3_f erosion intensified, revealing prominent needle-like protrusions on the surface (Figure 5(c_3_)). Based on their distinctive morphology and location, these features are interpreted as Al_18_B_4_O_33_ whiskers formed by the reaction between Al_2_O_3_ and the TiB_2_ oxidation product B_2_O_3_ [45]. H5 EDS analysis (Figure 6(b_2_)) indicates that this region primarily contains oxygen, silicon, and zirconium elements. Combined with Figure 6(b_1_), it is inferred that the main component is ZrSiO_4,_ consistent with the conclusion that both TiO_2_ and ZrSiO_4_ oxides coexist in the same sample.

The fracture surface of H10 (Figure 5(d_1_)) reveals more severe adhesion between Al_2_O_3_f, with multiple fibers fused and blurred boundaries, significantly compromising its load-bearing capacity. High-magnification images (Figure 5(d_2_,d_3_)) reveal accelerated surface erosion of the fibers, resulting in reduced diameters. Coral-like oxides consistent with the sample surface morphology (Figure 4(f_2_)) are observed in Figure 6(c_1_). EDS confirms their primary composition as ZrSiO_4_, matching the analysis results of the coral-like ceramic oxides in Figure 4(f_2_).

The fracture surface of H15 (Figure 5(e_1_)) demonstrates considerable debonding between the fibers and the matrix, indicative of further deterioration of the interfacial bonding. This delamination failure mode correlates with the jagged load–displacement curve observed in H15 (Figure 3f). Progressive erosion of the fibers is evident (Figure 5(e_1_,e_2_)), characterized by denser needle-like protrusions on the surface and the near-complete disappearance of fiber boundaries. Additionally, a lamellar structure consisting of multiple layers has been observed at the fiber intersections (Figure 5(e_2_,e_3_)), tentatively identified as metastable Al_4_B_2_O_9_ based on its morphology and growth environment [46]. This morphology is driven by two factors: the inherent two-dimensional growth preference of Al_4_B_2_O_9_ and the spatially limited environment in which it forms. The confined environment initially induces the formation of Al_4_B_2_O_9_ while inhibiting its transformation into the stable needle-like Al_18_B_4_O_33_ [47]. Furthermore, Figure 6(d_1_) shows elongated and coral-like oxides, with EDS confirming their primary composition as TiO_2_ and ZrSiO_4_.

After 20 heat treatments, the Al_2_O_3_f structure of H20 severely deteriorated (Figure 5(f_1_)), exhibiting extreme inter-fiber fusion and increased multilayered flaky material at the interfaces.

After 20 thermal cycles, the Al_2_O_3_f structure in H20 underwent severe degradation (Figure 5(f_1_)), characterized by extensive fiber fusion and an increased accumulation of multilayered lamellar deposits at the intersections of longitudinal and transverse fibers.

Needle-like Al_18_B_4_O_33_ whiskers further grew (Figure 5(f_2_)), with transverse fibers nearly losing their original shape entirely. Extended multiple heat treatments resulted in the deterioration of the mechanical support capability of the Al_2_O_3_f reinforcement, leading to a reduction in the flexural strength of H20 to 12.9 MPa (Figure 3e). High-magnification imaging (Figure 6(e_1_)) reveals the oxide region surface covered with more significant needle-like protrusions, whose morphology matches that of Al_18_B_4_O_33_ whiskers. EDS analysis (Figure 6(e_2_)) shows a strong characteristic aluminum peak at 1.5 keV, in addition to the major elements labeled in the Figure, further validating the inference that the needle-like structures are Al_18_B_4_O_33_ whiskers.

### 3.5. High-Temperature Phase Evolution and Thermodynamic Analysis

**Figure 7:**
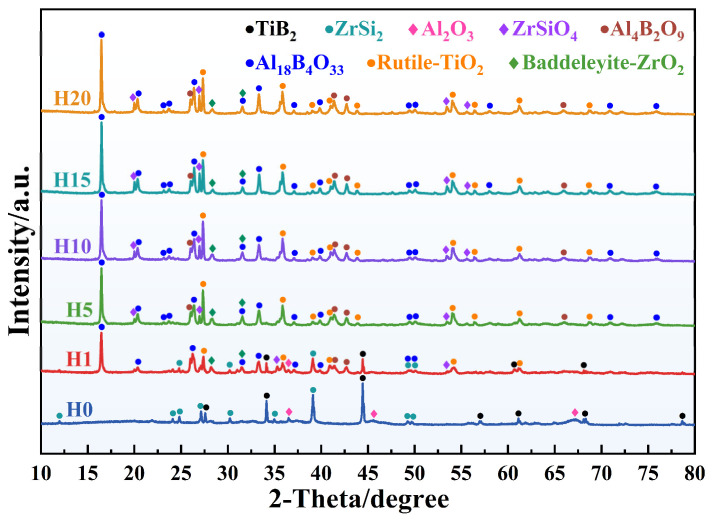
X-ray diffraction patterns of Z_20_T_25_ at different heat treatment cycles.

**Figure 8:**
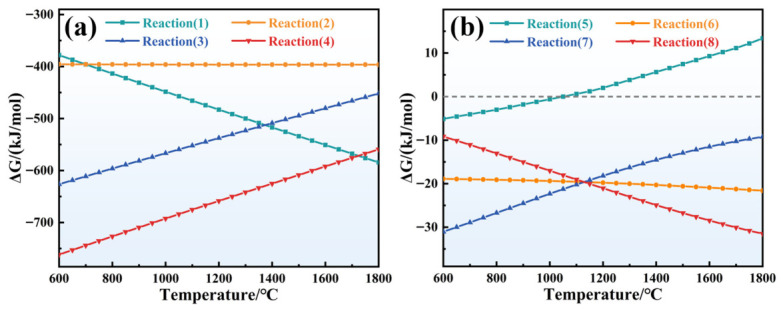
Gibbs free energy of key reactions during heat treatment. (**a**) Reactions (3)–(6), (**b**) Reac-tions (7)–(10).

To elucidate the phase evolution and thermodynamic reaction pathways of ceramizable composites during heat treatment, this research employed X-ray diffraction (XRD) for phase characterization of a series of samples (Figure 7). The Gibbs free energy change (ΔG) for relevant reactions was calculated using HSC Chemistry software (6.0), with results presented in Figure 8.

The XRD results indicate that untreated Z_20_T_25_ (H0) primarily comprises ZrSi_2_ and TiB_2_ crystalline phases, in addition to trace quantities of Al_2_O_3_. During high-temperature heat treatment, BPR pyrolysis produces substantial amounts of PyC, which are readily oxidized in an environment of high temperature and oxygen. HSC calculations demonstrated that at 1200 °C, both Reactions 3 and 4 exhibited negative ΔG values, with Reaction 3 displaying a larger magnitude of ΔG. According to the Gibbs free energy criterion, the principal gaseous product resulting from PyC oxidation should be CO.

Following a single heat treatment (H1), the diffraction peak intensities of the original phases, such as ZrSi_2_ and TiB_2_, were significantly reduced; however, they did not entirely disappear, indicating partial transformation into more stable oxides or silicates. Concurrently, new diffraction peaks corresponding to rutile-type TiO_2_ and zircon-type ZrO_2_ emerged in the H1 spectrum, thereby further substantiating the oxidation of TiB_2_ and ZrSi_2_. This observation aligns with the SEM data (Figure 4(b_2_)), which suggests TiO_2_ as the predominant oxide component. TiB_2_ undergoes oxidation to produce TiO_2_ and B_2_O_3_ (Reaction 5), while ZrSi_2_ oxidizes to yield ZrO_2_ and SiO_2_ (Reaction 6). Thermodynamic assessments reveal that the ΔG values for both Reactions 5 and 6 are significantly negative, thereby confirming their high spontaneous propensity. Notably, while ZrO_2_ was detected, no diffraction peaks for SiO_2_ were observed; this is due to the rapid cooling of the post-heat-treated sample at room temperature, which inhibits SiO_2_ crystallization and results in an amorphous glass phase. Furthermore, the diffraction peaks for ZrSiO_4_ appeared in the H1 spectrum, originating from the subsequent reaction between amorphous SiO_2_ and ZrO_2_ to form ZrSiO_4_ (Reaction 7). Although the ΔG for Reaction 7 at 1200 °C is marginally positive, the reaction becomes spontaneous during cooling to ambient temperature. Consequently, ZrSiO_4_ was formed during H1, with its diffraction peak intensity increasing in proportion to the number of heat treatments.

2C + O_2_(g) = 2CO(g) (3)

C + O_2_(g) = CO_2_(g) (4)

2/5TiB_2_ + O_2_(g) = 2/5TiO_2_ + 2/5B_2_O_3_ (5)

1/3ZrSi_2_ + O_2_(g) = 1/3ZrO_2_ + 2/3SiO_2_ (6)

ZrO_2_ + SiO_2_ = ZrSiO_4_ (7)

Notably, the most prominent diffraction peak is observed near 2θ = 16.5°. Systematic analysis and comparative studies confirm that this position corresponds to the diffraction peak of Al_18_B_4_O_33_. At a temperature of 1200 °C, B_2_O_3_ exists in a liquid phase and demonstrates high volatility, thereby enabling complete contact with the Al_2_O_3_ contained within the fibers. Consequently, Al_2_O_3_ reacts with B_2_O_3_ to produce significant quantities of Al_18_B_4_O_33_ (Reaction 8). As the number of heat treatment cycles increases, Reaction 8 proceeds continuously, thereby intensifying the chemical erosion of Al_2_O_3_ fibers. Simultaneously, a minor diffraction peak corresponding to Al_4_B_2_O_9_ (Reaction 9) was observed in the pattern, consistent with the presence of a small amount of multilayered flaky material in the fracture surface SEM. Thermodynamic calculations demonstrate that the ΔG values for Reactions 8 and 9 are comparable and both exhibit negative values. However, Al_4_B_2_O_9_ is a metastable phase that can be transformed into the thermodynamically stable Al_18_B_4_O_33_ at high temperature through Reaction 10. Therefore, the system ultimately produces a substantial quantity of Al_18_B_4_O_33_, with only a minor residual amount of Al_4_B_2_O_9_ remaining.

Al_2_O_3_ + 2/9B_2_O_3_ = 1/9Al_18_B_4_O_33_ (8)

Al_2_O_3_ + 1/2B_2_O_3_ = 1/2Al_4_B_2_O_9_ (9)

Al_2_O_3_ + 2/5Al_4_B_2_O_9_ = 1/5Al_18_B_4_O_33_ (10)

In the XRD patterns of the H5 sample, the diffraction peaks of the original phases, such as ZrSi_2_ and TiB_2_, have completely disappeared, indicating their complete conversion into more stable oxide or silicate phases. Simultaneously, the diffraction peaks of oxidation products like Al_18_B_4_O_33_ and TiO_2_ have further intensified, suggesting that compared to H1, fiber erosion has intensified, and the proportion of oxidation products within the system has significantly increased. In the subsequent series of heat-treated samples (H10–H20), the diffraction patterns reveal no shifts in phase peak positions while the diffraction peaks continue to intensify. This phenomenon demonstrates that the crystal structure and chemical composition of the composite remain stable throughout repeated thermal cycles, showcasing its broad application potential in reusable systems.

### 3.6. In Situ Ceramization Process and the Failure Mechanism

**Figure 9:**
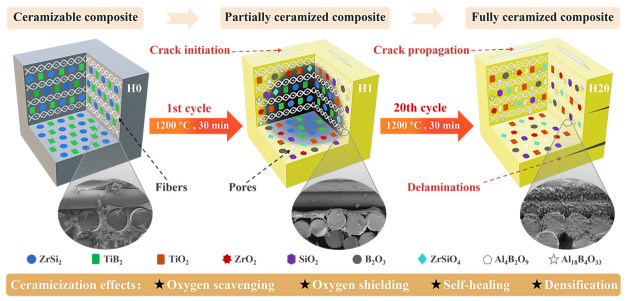
In situ ceramization process and failure mechanism of ceramizable composites.

Through a comprehensive multidimensional analysis encompassing thermal properties, flexural strength evolution, microstructure (surface and fracture surface), phase composition, and thermodynamic calculations, this research elucidates the in situ ceramization process and failure mechanism of ceramizable composites (Figure 9).

During heat treatment at temperatures of 1200 °C, the BPR in the Z_20_T_25_ sample undergoes pyrolysis, releasing volatile gases and forming PyC. Concurrently, the functional fillers ZrSi_2_ and TiB_2_ undergo passive oxidation, resulting in the in situ formation of oxides such as TiO_2_, B_2_O_3_, ZrO_2_, and SiO_2_. This process yields a significant synergistic protective effect: on one hand, the oxidation of ceramic fillers consumes oxygen, thereby reducing its partial pressure and delaying both BPR pyrolysis and PyC oxidation; on the other hand, the generated oxides form protective layers on the surfaces of fibers and the matrix, hindering further oxygen attack. Significantly, the low-viscosity liquid phase produced by B_2_O_3_ and SiO_2_ at high temperatures flows viscously, filling internal pores and microcracks, thus promoting self-healing and densification. The integration of these protective mechanisms enables the composite to maintain substantial mechanical load-bearing capacity and high mass even after prolonged exposure to high temperatures.

Nevertheless, as the number of heat treatment cycles progresses, the ongoing pyrolysis of BPR and the extensive oxidation of PyC inflict significant damage upon the matrix, resulting in surface crack propagation and a reduction in interlayer bonding strength. More detrimentally, the liquid phase of B_2_O_3_ interacts at the interface with Al_2_O_3_f, leading to the formation of Al_18_B_4_O_33_ and Al_4_B_2_O_9_, which causes chemical corrosion and structural deterioration of the reinforcing fibers, thereby considerably impairing their load-bearing capacity. Furthermore, as B_2_O_3_ continuously volatilizes and reacts with Al_2_O_3_, the pore-filling and self-healing properties of the liquid phase gradually decline, thereby increasing the internal porosity of the material. Ultimately, under the combined effects of interlaminar performance degradation, damage to the fiber skeleton, and the accumulation of pore defects, the composite undergoes severe damage or even fails.

## 4. Conclusions

This research utilized a pre-impregnated hot-pressing process to produce a novel ZrSi_2_-TiB_2_-modified alumina fiber/boron phenolic resin ceramizable composite. The principal findings are summarized as follows: The composite exhibits exceptional thermal insulation performance, with a low thermal conductivity of 0.405 W·m^−1^·K^−1^. It also demonstrates superior thermal stability, maintaining a mass retention rate of 89.45% after exposure to 1200 °C in an air atmosphere. The composite displays remarkable reusability under extreme thermal conditions. After 20 heat treatments at 1200 °C (totaling 600 min), the dimensional change rate in both length and width remains below 0.6%, with mass retention exceeding 80%. Following 15 heat cycles, the flexural strength remained above 20 MPa, indicating its potential to fulfill service requirements for reusable materials. The enhanced material performance originates from the synergistic in situ ceramization of ZrSi_2_ and TiB_2_. These ceramic fillers protect the carbon matrix through a combined oxygen-consumption and oxygen-barrier mechanism. More importantly, the high-temperature liquid phase derived from the ceramic oxidation products, mainly SiO_2_ and B_2_O_3_, undergoes viscous flow, thereby enabling pore self-healing and matrix densification. This process preserves the load-bearing capacity of the material and significantly improves the reusability of the composite. With increasing heat-treatment cycles, B_2_O_3_ continuously volatilizes at high temperatures and is further consumed through reactions with Al_2_O_3_. As a result, the self-healing effect of the high-temperature liquid phase gradually weakens, accompanied by an increase in porosity. Ultimately, the combined effects of interlaminar-property degradation, damage to the fiber skeleton, and accumulated pore defects lead to a substantial decline in the load-bearing capacity of the composite. In conclusion, the ceramizable composite developed herein demonstrates broad potential for applications in reusable thermal protection materials, owing to its excellent thermal insulation, oxidation resistance, dimensional stability, and reusability.

## Figures and Tables

**Table 1:** Ratio of ceramizable composites.

Samples	Weight/phr				
	Al_2_O_3_f	BPR	EtOH	ZrSi_2_	TiB_2_
Z_0_T_0_	100	100	100	0	0
Z_20_T_25_	100	100	100	20	25

**Table 2:** Thermal conductivity of ceramizable composites.

Samples	Thermal Conductivity/W·m^−1^·K^−1^					Average Value /W·m^−1^·K^−1^
	1	2	3	4	5	
Z_0_T_0_	0.273	0.264	0.265	0.266	0.272	0.268
Z_20_T_25_	0.421	0.419	0.418	0.384	0.385	0.405

**Table 3:** *LAR*, *MAR*, and backface temperature of composites.

Samples	4186 kW·m^−2^, 30 s			1500 kW·m^−2^, 60 s		
	*LAR* /mm·s^−1^	*MAR* /g·s^−1^	Backface Temperature /°C	*LAR* /mm·s^−1^	*MAR* /g·s^−1^	Backface Temperature /°C
Z_0_T_0_	0.1350 ± 0.0022	0.0652 ± 0.0028	40.1 ± 1.8	0.0343 ± 0.0017	0.0502 ± 0.0057	65.8 ± 3.3
Z_20_T_25_	0.1363 ± 0.0019	0.0570 ± 0.0013	52.9 ± 1.4	0.0317 ± 0.0020	0.0198 ± 0.0022	71.0 ± 3.1

## Data Availability

The data are available from the corresponding author upon reasonable request.
